# LightECA-UNet: a lightweight model for segmentation of coal fracture CT images

**DOI:** 10.1038/s41598-026-37291-7

**Published:** 2026-01-23

**Authors:** Xiaoyu Xing, Yingying Li, Yimin Zhang, Huanli Li, Guoqiang Wang

**Affiliations:** 1 School of Computer Science, Luoyang Institute of Science and Technology, Luoyang, 471023 China; 2 State Key Laboratory Cultivation Base for Gas Geology and Gas Control, Henan Polytechnic University, Jiaozuo, 454000 China

**Keywords:** Coal CT images, Fracture segmentation, Lightweight convolutional neural network, Efficient channel attention, Depthwise separable convolution, Energy science and technology, Engineering, Solid Earth sciences

## Abstract

Coal fracture segmentation in CT images is critical for coal structure analysis, coalbed methane extraction, and mine safety, but it is challenged by complex fracture features and limited computing resources for mine-site deployment. Basic UNet exhibits redundancy, sensitivity to image noise, and high overfitting risk. This study proposes LightECA-UNet, integrating depthwise separable convolution (DSC), efficient channel attention (ECA), and adaptive channel pruning. Experiments show LightECA-UNet achieves 1.6% higher mean Intersection over Union (mIoU) and 2.5% higher fracture IoU than currently popular models. Compared to lightweight counterparts, it reduces computational load by 87.1% and parameter count by 86.9%, enabling deployment on mine-used edge equipment while maintaining segmentation accuracy.

## Introduction

Coal fractures regulate coal-rock mechanical properties and fluid migration, and their morphological parameter quantification provides critical support for coal stability evaluation and early warning of water inrush and gas outburst in mining engineering. Computed Tomography (CT) technology, leveraging its non-destructive advantage, has become the primary means to acquire 3D internal fracture information of coal. Relevant advances include: Ramandi et al. (2016) realized quantitative characterization of coal porosity and permeability via micro-CT, establishing a direct link between CT-derived parameters and seepage properties^1^; Liu et al. (2017) combined FIB-SEM and X-ray CT to reveal interconnected pore characteristics in high-rank coal, overcoming the limitation of single technology in resolving multi-scale structures^2^; Roslin et al. (2019) integrated micro-CT and SEM for coal cleat structure analysis and permeability simulation, providing technical support for fluid flow prediction^3^; Jing et al. (2020) constructed a hybrid fracture-micropore network model to simulate multiphysics gas flow in coal, laying a foundation for optimizing gas extraction schemes^4^. However, coal CT fracture segmentation faces two intractable bottlenecks: blurred boundaries due to similar grayscale between fractures and matrix, and poor real-time inference of high-resolution images—both severely restricting CT technology’s engineering application in coal fracture research.

The rise of large vision models (LVMs) such as the Segment Anything Model (SAM) has opened new avenues for geoscientific imaging. Li et al. (2025) enhanced and assessed LVMs for 3D particle reconstruction from X-ray tomography, demonstrating their potential in processing CT-derived geological data by improving feature extraction from low-contrast structures^5^; Li et al. (2025) further utilized vision foundation models for 3D reconstruction of granular media, verifying LVMs’ adaptability to geological material characterization^6^. Despite these advances, LVMs’ heavy parameters and natural image pre-training limit their direct deployment on mine-used equipment. Prior to LVMs, coal fracture segmentation relied on traditional methods and task-specific deep learning, both with limitations: Zhang et al. (2013) used the Hessian matrix for multi-scale segmentation of coal piles on belts, but grayscale fluctuations at coal-rock interfaces restricted connectivity identification accuracy^7^; Xue et al. (2019) proposed an FCN-based algorithm for rock concrete crack geometry identification, yet its single-scenario training dataset caused poor generalization in mineral-mixed coal images^8^.

Semantic segmentation breakthroughs laid a theoretical foundation for addressing these issues. Ronneberger et al. (2015) proposed UNet with an “encoder-decoder + skip connection” structure, fusing shallow edge details and deep semantic features to resolve traditional models’ “detail loss” and “semantic ambiguity”^9^. Subsequent advances optimized model performance from multiple dimensions: Howard et al. (2017) proposed MobileNets for lightweight design via depthwise separable convolution^10^; Huang et al. (2017) developed DenseNet to enhance feature reuse through dense connections^11^; Sandler et al. (2018) optimized MobileNetV2 with inverted residuals and linear bottlenecks^12^; Hu et al. (2018) pioneered the channel attention mechanism in SE-Net^13^; Oktay et al. (2018) integrated attention into UNet to improve weak feature capture^14^; Han et al. (2020) proposed GhostNet to generate more features with cheap operations, further advancing lightweight design^15^. Beyond general-purpose advancements, domain-specific segmentation has demonstrated the value of scenario-adaptive model design: Zheng et al. (2024) developed Coralscop to achieve accurate segmentation of arbitrary coral images by customizing the architecture for coral-specific textures^16^; Zhang et al. (2024) proposed CNet for seabed coral reef segmentation, verifying that tailoring models to target scene characteristics effectively improves segmentation performance^17^. These technical accumulations, both general and domain-specific, provide solid support for adapting segmentation models to coal scenarios.

With UNet’s popularization, coal fracture segmentation entered the deep learning era. Liu et al. (2019) proposed a multi-scale feature fusion method for coal-rock recognition based on completed local binary pattern and CNN^18^; Diakogiannis et al. (2020) developed ResUNet-a for semantic segmentation of remotely sensed data, which was later adapted for coal fracture segmentation^19^; Lu et al. (2020) proposed an adaptive multi-scale feature fusion residual U-Net to optimize coal-rock image feature extraction^20^; Karimpouli et al. (2020) systematically verified the feasibility of CNNs in coal cleat/fracture segmentation^21^; Wang et al. (2020) proposed ECA-Net, a lightweight channel attention mechanism using 1D convolution, providing a new idea for low-contrast fracture segmentation^22^; Liu et al. (2021) designed a parallel attention UNet for crack detection, enhancing feature focus capability^23^; Lu et al. (2021) further improved U-Net with adaptive multi-scale feature fusion to boost coal-rock fracture segmentation accuracy, but ignored computing power constraints^24^; Wang et al. (2022) applied an improved U-Net to outcrop fracture segmentation, verifying its field applicability but lacking micro-fracture optimization^25^; Ali et al. (2022) deployed a modified U-Net on edge devices, confirming the necessity of model compression but suffering significant accuracy loss^26^; Rahman et al. (2025) proposed BiSeNet-CBAM for real-time semantic segmentation, though it was not designed for coal’s low-contrast features^27^; Dong et al. (2024) developed a DC-UNet-based method for detecting fractures along coal mine roadway Sect^28^.; Wang et al. (2024) realized intelligent extraction of coal micro-CT fissures via deep learning^29^; Wang et al. (2025) proposed the VRA-UNet network for fracture identification and 3D reconstruction of coal-rock combinations^30^; Jin et al. (2025) designed a crack segmentation model for coal-rock CT images based on explicit visual prompting^31^. Despite these progresses, balancing model lightweight, accuracy, and scenario adaptability remains unresolved for coal CT fracture segmentation.

To address these gaps, this study proposes LightECA-UNet, a lightweight coal CT fracture segmentation architecture optimized from traditional UNet. Its core design targets mine equipment constraints: replacing double convolution modules with Depthwise Separable Convolution (DSC) to reduce computational redundancy, introducing targeted channel pruning (halving channels in the encoder’s first downsampling layer and decoder’s last upsampling layer) to shrink model size, and integrating the Efficient Channel Attention (ECA) mechanism after DSC to compensate for channel isolation—enhancing sensitivity to low-contrast fractures without extra computational branches.

The main contributions of this paper are as follows:

1. VGG-Free Architecture Reform: Proposes a VGG-free U-shaped architecture to eliminate the inherent incompatibility between the VGG16 backbone and DSC in existing methods, laying a foundational framework for lightweight deployment on mine-used equipment.
2. Channel Pruning and Accuracy Balance Rules: Develops a scenario-specific lightweight strategy that halves the number of channels in key layers, achieving effective model compression while avoiding excessive accuracy loss.
3. ECA-DSC Symbiotic Block: Constructs a novel symbiotic block by placing local-cross-channel ECA attention after DSC’s depthwise convolution, compensating for channel isolation without adding extra branches.
4. Dual Optimization of Efficiency and Accuracy: Under the premise of significantly reducing computational load and model size, the improved LightECA-UNet still achieves a slight but considerable increase in segmentation accuracy, providing a practical engineering solution for coal fracture segmentation.

## Model architecture

Fracture segmentation in coal CT images is a core technology for the quantitative analysis of coal structure, with its core challenge lying in the contradiction between the complexity of fracture features and the computational efficiency of high-resolution images. In CT images, coal fractures exhibit characteristics of variable scales, blurred edges, and small gray scale differences from the background; some micro-fractures are only a few pixels wide, while high-resolution images impose strict requirements on the model’s computing power. The annotated sample library has been constructed through CT scanning and manual annotation to provide data support for model training. However, the basic UNet has significant limitations in this task: traditional convolution leads to computational redundancy; the lack of a feature selection mechanism makes it vulnerable to noise interference; and fixed channel configuration causes parameter redundancy and overfitting. To this end, this paper proposes a lightweight architecture, LightECA-UNet, which achieves the balance between segmentation accuracy and computational efficiency by optimizing feature extraction, enhancing key feature responses, and simplifying parameters.

### Overview of the overall structure

The encoder-decoder symmetric structure and skip connection of UNet provide a foundation for fracture segmentation. The encoder extracts high-level semantic features through downsampling max-pooling, while the decoder restores spatial resolution through upsampling and fuses shallow and deep features. However, the direct application of the basic UNet to coal CT image fracture segmentation has three limitations: The computational load of 3 × 3 traditional convolution increases multiplicatively with the product of the number of channels and the size of the feature map, resulting in low processing efficiency for high-resolution images; All channel features are treated uniformly without distinguishing the feature responses between fractures and background noise; The fixed channel growth strategy (64–128-256–512) is not adapted to the characteristic of limited gray scale variation range of coal fractures, easily leading to parameter redundancy and overfitting.​.

**Fig. 1:**
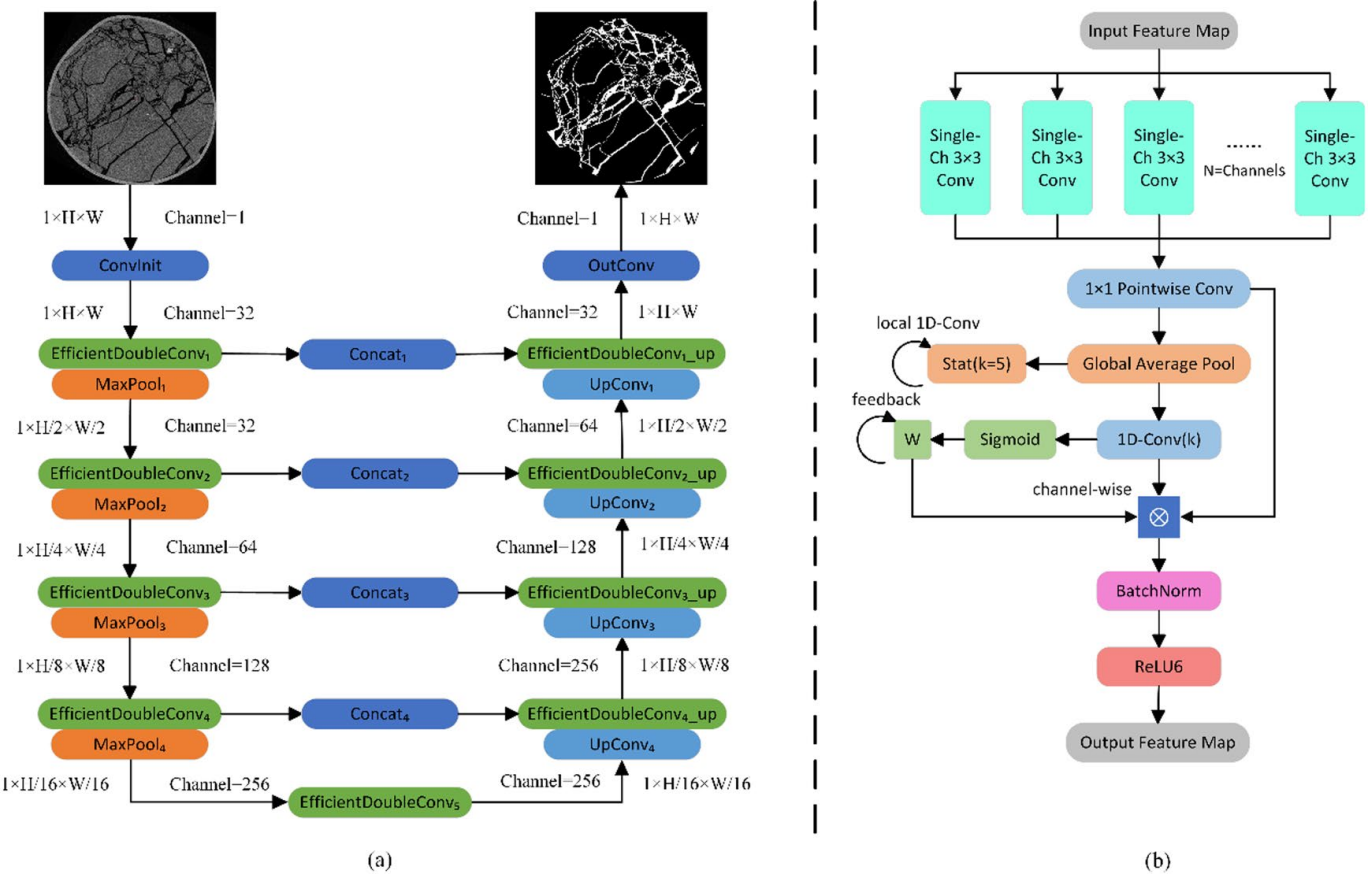
Schematic diagram of the overall framework (**a**), schematic diagram of EfficientDoubleConv (**b**).

LightECA-UNet addresses the above problems through three core improvements: It adopts depthwise separable convolution instead of traditional convolution, decomposing the processes of spatial feature extraction and channel fusion to reduce the computational load; It embeds the ECA attention mechanism to enhance fracture feature responses by adaptively learning channel weights; It adjusts the encoder-decoder channel scale according to the statistical characteristics of coal fractures to construct a lightweight feature space. The optimized model retains the advantages of the UNet symmetric structure and skip connection, effectively adapting to the needs of coal CT image fracture segmentation. The overall framework is shown in Fig. 1a, and the EfficientDoubleConv is shown in Fig. 1b.

### Depthwise separable convolution (DSC)

The basic UNet employs a DoubleConv structure comprising two convolutional layers, batch normalization, and an activation function for feature extraction. The computational load of its 3 × 3 convolution is expressed as:

1 $$\:{N}_{compute}={W}_{out}\times\:{H}_{out}\times\:{K}^{2}\times\:{C}_{in}\times\:{C}_{out}$$

Where$\:\:\:{\mathrm{W}}_{\mathrm{o}\mathrm{u}\mathrm{t}}\:\:$and $\:{\mathrm{H}}_{\mathrm{o}\mathrm{u}\mathrm{t}}$ are the width and height of the output feature map dimensions, $\:\mathrm{K}$ the kernel size, ​$\:{\mathrm{C}}_{\mathrm{i}\mathrm{n}}$ and ​$\:{\mathrm{C}}_{\mathrm{o}\mathrm{u}\mathrm{t}}$ the input and output channel counts.

For a 512 × 512 single-channel CT image processed through the first DoubleConv layer expanding from 1 to 32 channels, a single convolution requires approximately 755 million operations. This substantial computational complexity constrains model efficiency.

**Fig. 2:**
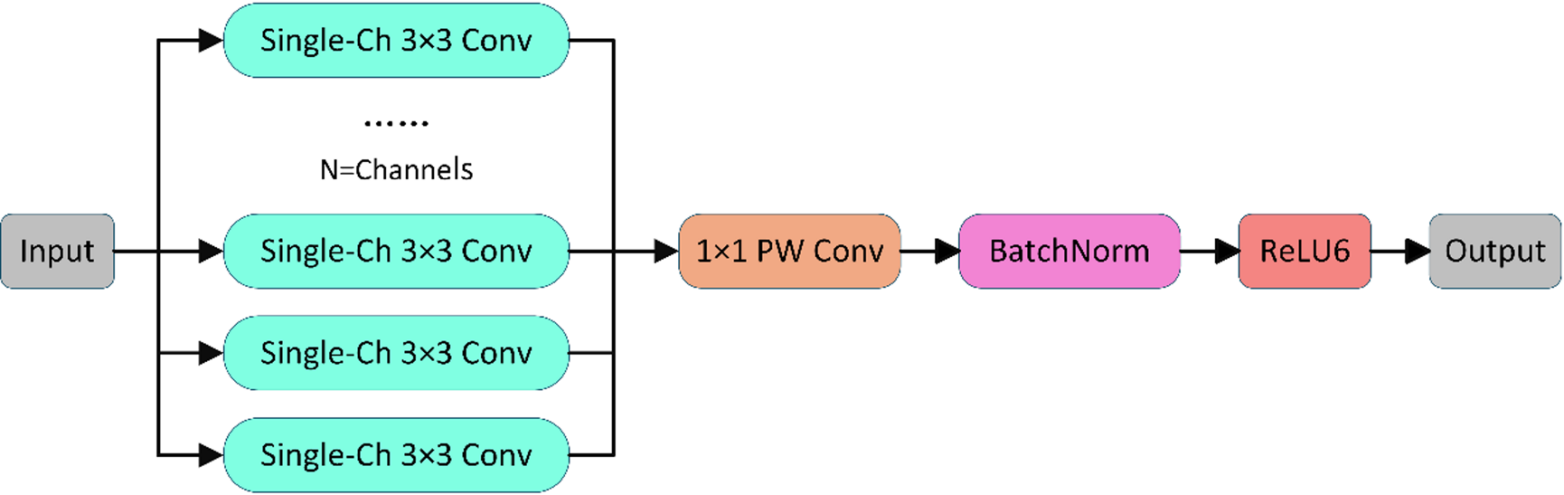
Framework diagram of the depthwise separable convolution module.

To address this bottleneck, the method proposed in this paper adopts depthwise separable convolution as a replacement for the conventional double convolution module, and decomposes the convolution operation into two successive steps, namely depthwise convolution and pointwise convolution. This separation of spatial feature extraction and channel fusion significantly reduces computational load. The overall framework diagram of the module is shown in Fig. 2.

The total computational load of depthwise separable convolution is the sum of the computational loads of depthwise convolution and pointwise convolution, where:​.

The computational load formula of depthwise convolution is:

2 $$\:{N}_{depthwise}={W}_{out}\times\:{H}_{out}\times\:{K}^{2}\times\:{C}_{in}$$

The computational load formula of pointwise convolution is:

3 $$\:{N}_{pointwise}={W}_{out}\times\:{H}_{out}\times\:{C}_{in}\times\:{C}_{out}$$

Therefore, the total computational load of depthwise separable convolution is:

$$\:{N}_{ds-compute}={N}_{depthwise}+{N}_{pointwise}$$

4 $$\:={W}_{out}\times\:{H}_{out}\times\:\left({K}^{2}\times\:{C}_{in}+{C}_{in}\times\:{C}_{out}\right)$$

In the EfficientDoubleConv module of LightECA-UNet, depthwise separable convolution is implemented through the DepthwiseSeparableConv class. Specifically, a 3× 3 convolution kernel is first applied to each input channel. This convolution kernel is responsible for extracting spatial features within a single channel, without involving information interaction between channels, and only performs local feature extraction within individual channels. As a result, the computational complexity is only related to the number of input channels, the size of the convolution kernel, and the size of the feature map.

After completing the depthwise convolution, a pointwise convolution operation is performed. In this step, a 1 × 1 convolution kernel is used to linearly combine the output channels of the depthwise convolution, enabling feature fusion between different channels. The number of 1 × 1 convolution kernels is consistent with the target number of output channels; each 1 × 1 convolution kernel performs a weighted summation of features across all input channels, thereby mapping the multi-channel features output by the depthwise convolution to the target channel dimension. Consequently, the computational complexity of the pointwise convolution is only related to the number of input channels, the number of output channels, and the size of the feature map.

By comparing the ratio of formula (1) to formula (4), the lightweight advantage of depthwise separable convolution can be clearly reflected:

5 $$\:\frac{{N}_{ds-compute}}{{N}_{compute}}=\frac{{K}^{2}\times\:{C}_{in}+{C}_{in}\times\:{C}_{out}}{{K}^{2}\times\:{C}_{in}\times\:{C}_{out}}=\frac{1}{{C}_{out}}+\frac{1}{{K}^{2}}$$

When the output channel number$\:\:{C}_{out}=32$ and the convolutional kernel size $\:K=3$, this ratio is approximately $\:\frac{1}{32}+\frac{1}{9}\approx\:\frac{1}{9}$, meaning the computational load of depthwise separable convolution is only about $\:\frac{1}{9}$ of that of traditional convolution. This significant reduction in computational load allows the model to maintain spatial feature extraction capability while processing high-resolution coal CT images, greatly reducing computational consumption and improving training and inference efficiency. For example, for the previously mentioned $\:512\times\:512$CT image, after replacing traditional convolution with depthwise separable convolution, substituting into formula (4) gives the computational load of the first DoubleConv layer as $\:512\times\:512\times\:\left({3}^{2}\times\:1+1\times\:32\right)=1.07\times\:1{0}^{8}$ operations, reduced from about 755 million to 107 million. This effectively solves the computational bottleneck of traditional convolution, making it possible for the model to process large-scale CT image data.

### ECA attention mechanism

In coal CT images, microfractures and the surrounding coal matrix exhibit significant grayscale overlap, making direct differentiation based on grayscale features alone challenging. Meanwhile, fracture feature responses vary substantially across different channels. High-frequency features that characterize fracture edges are concentrated in specific channels, whereas low-frequency background information is distributed across other channels. Traditional UNet treats all channels equally without prioritizing key channels related to fractures. Depthwise separable convolution further weakens the effectiveness of spatial skip connections by isolating channel-wise information interaction. These factors collectively reduce the model’s ability to capture low-contrast fracture features.

**Fig. 3:**
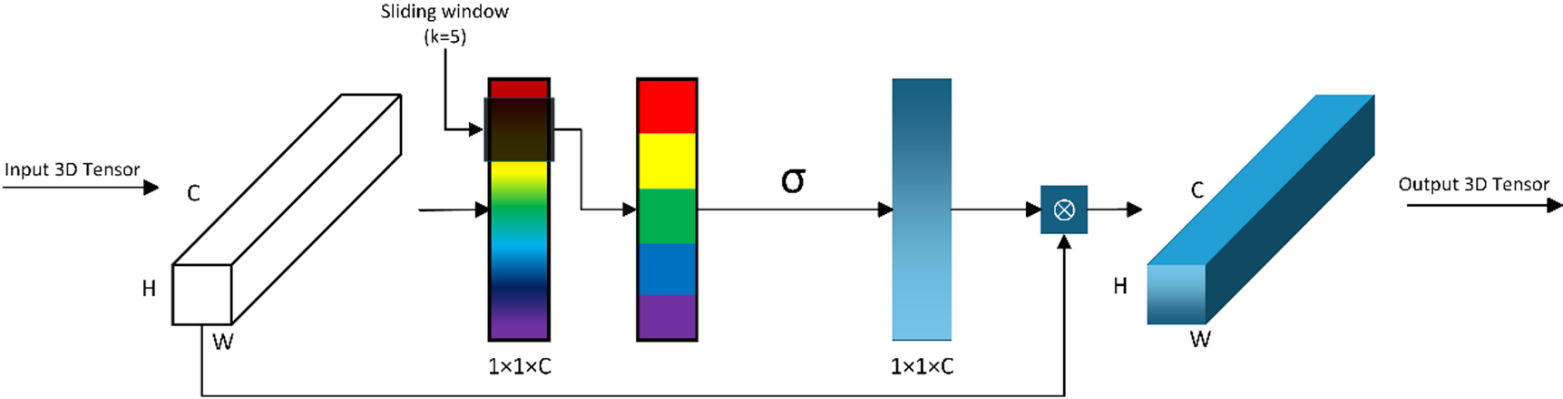
Schematic diagram of ECA module.

The Efficient Channel Attention mechanism addresses these challenges through a lightweight and locally adaptive design, making it well-suited for coal CT fracture segmentation. Its core innovation lies in modeling channel correlations via local cross-channel interaction. This approach avoids the parameter redundancy of fully connected layers in traditional attention mechanisms while maintaining sensitivity to weak fracture features. The architecture of the ECA module is shown in Fig. 3.

The workflow of the ECA mechanism consists of four key steps, with design choices tailored to the characteristics of coal CT images:

First, global average pooling compresses the 2D feature map of each channel into a scalar statistical value. This operation averages all pixels in the H×W feature map of each channel to capture global feature information. It effectively suppresses local noise interference common in coal CT images, such as grayscale fluctuations caused by scanning artifacts. By reducing high-dimensional feature maps to low-dimensional scalars, it also lays a foundation for efficient channel correlation modeling without losing critical fracture feature trends.

Second, adaptive kernel size calculation dynamically determines the 1D convolutional kernel size based on the number of channels. Kernel size directly affects the range of channel correlation modeling. A kernel that is too small can only capture correlations between adjacent channels, failing to integrate scattered fracture features across nearby channels. A kernel that is too large introduces irrelevant background channel noise, diluting the response of fracture-related channels. The ECA mechanism uses the following formula to calculate the kernel size:

6 $$\:k=\psi\:\left(C\right)=\lfloor\:\left|{{log}}_{2}(C\right)/\gamma\:|+b{\rfloor\:}_{odd}$$

Hyperparameters γ and b are set to 2 and 1 respectively through iterative optimization for the coal CT fracture segmentation task. This setting balances correlation coverage and noise suppression. For instance, when the number of channels reaches a value common in middle encoder layers, the calculated kernel size matches the local distribution of fracture features. When the number of channels increases to a value typical of deep encoder layers with more complex features, the kernel size expands appropriately to enable broader but targeted correlation modeling. This adaptive adjustment ensures that the ECA module can adapt to varying channel dimensions throughout the network, maintaining consistent sensitivity to low-contrast fractures.

Third, 1D convolution captures local dependencies between channels. The input to this step is the sequence of channel statistical values generated by global average pooling. Symmetric padding is applied during convolution to preserve the sequence length, ensuring that each output value is a weighted sum of the input sequence and its adjacent channels. This local convolution focuses on correlations between nearby channels, aligning with the spatial continuity of fracture features in coal CT images. It effectively enhances the aggregation of weak fracture features across adjacent channels while avoiding interference from distant background channels.

Finally, attention weights are generated and applied to the original feature map. The outputs of the 1D convolution are mapped to the range [0,1] using a Sigmoid activation function, producing per-channel attention weights. Higher weights are assigned to channels containing fracture features, such as edge-related high-frequency channels. Lower weights suppress channels dominated by background or noise. These weights are then multiplied with the original feature map in a channel-wise manner, directly enhancing the response of fracture features and weakening background interference.

This design of the ECA mechanism offers two key advantages for coal CT fracture segmentation. First, it avoids the computational overhead of spatial attention branches, making it compatible with the lightweight requirement of mine-edge deployment. Second, it maintains high sensitivity to low-contrast features by preserving channel-wise information integrity and adapting to local feature distributions. This ensures the ECA mechanism can effectively enhance the discriminability of fracture features in coal CT images without compromising the model’s efficiency.

### Adaptive channel number adjustment

Coal CT image fracture segmentation differs from general semantic segmentation in feature traits. Fracture grayscale variation is confined to a narrow range near the coal matrix value, and fracture pixels account for a small proportion of the entire image. High-dimensional feature spaces, necessary for complex texture differentiation in natural images, are unnecessary here. Excessive channels increase model parameters and computational complexity, raise overfitting risks under limited datasets, and impede shallow-deep feature integration during skip connections, leading to fine fracture detail loss and reduced accuracy. The basic UNet adopts a fixed channel growth strategy that progresses from 64 to 128, 256 and finally 512 channels in the encoder. This strategy fails to adapt to the traits of coal CT fracture segmentation, causing severe parameter redundancy and inefficient feature utilization.

To address this, LightECA-UNet proposes an adaptive channel adjustment strategy tailored to coal CT fracture segmentation. Its core innovation lies in constructing a lightweight feature space by pruning redundant channels based on coal fracture feature statistics, while ensuring sufficient feature representation. This strategy breaks traditional UNet’s rigid channel doubling logic and balances lightweight performance with segmentation accuracy.

In the encoder, initial channels are reduced from 64 to 32. This adjustment aligns with the narrow grayscale range of coal CT images. Thirty-two channels suffice to capture initial fracture features such as edge contours and grayscale differences, while cutting input layer computation and parameters by nearly half. Subsequent downsampling adopts a 32–64-128–256-256 growth strategy. Channels double to 64 after the first downsampling, increase to 128 after the second, reach 256 after the third, and remain 256 after the fourth. Stopping channel doubling at the fourth downsampling is determined by deep-layer feature complexity. Deep layers focus on high-level semantic features such as fracture connectivity, and channels beyond 256 add no discriminative value but introduce redundancy.

For the decoder, a symmetric 256-128-64-32-32 reduction strategy matches encoder feature dimensions during skip connections. Each upsampling uses transposed convolution to halve channels, followed by concatenation with corresponding encoder features and compression to target dimensions via EfficientDoubleConv. For instance, 256-dimensional deep features are reduced to 128 channels in the first upsampling, concatenated with 256-dimensional encoder features to form 384 dimensions, and then compressed to 128 dimensions. This ratio optimizes coal fracture feature fusion, preserving edge details and semantic information. The final decoder layer retains 32 channels before a 1 × 1 convolution maps features to a single channel for the segmentation mask. This retention prevents detail loss of microfractures with only a few pixels in width.

7 $$\:{N}_{params}={K}^{2}\times\:{C}_{in}\times\:{C}_{out}+{C}_{out}$$

where the second term denotes bias parameters. When batch normalization is included, an additional$\:4\times\:{C}_{out}$parameters are added, including mean, variance, scale factor and shift factor. By this formula, LightECA-UNet’s parameter count is approximately 550,000, which is only 1/56 of basic UNet’s 3.1 million parameters. This reduction lowers overfitting risks and synergizes with depthwise separable convolution and the ECA attention mechanism. Fewer channels reduce the computational basis of depthwise separable convolution, while the ECA attention mechanism enhances feature utilization in constrained channels. This synergy ensures high segmentation accuracy while achieving extreme lightweight, adapting to mine-edge equipment deployment and the unique demands of coal CT fracture segmentation.

## Experiments and analysis

Building upon the systematic elaboration of the model architecture design, this chapter aims to rigorously validate the comprehensive performance of the proposed improved UNet model through experiments. To scientifically evaluate the model’s effectiveness in the coal CT image fracture segmentation task, a complete quantitative evaluation framework is established, employing multi-dimensional metrics for a thorough analysis. The experiments will first detail the specific environment configuration and data preparation plan, then introduce the definitions and calculation basis of each evaluation metric, and finally validate the model’s comprehensive performance in terms of accuracy and efficiency through systematic experimental results, providing a reliable algorithmic performance basis for coal industrial applications.

### Dataset preparation

This dataset is constructed from complete CT slices with complex structures obtained during coal seam fracturing experiments. Cylindrical raw coal samples with a diameter of 25 mm and a height of 50 mm were scanned without cropping or preprocessing, allowing direct segmentation of the original images. The resolution of the CT slices is approximately 20 μm, and the size of the finally output original images is about 1248 × 1248. A total of 600 images were selected for dataset construction, and the training set, validation set, and test set were split in a ratio of 7:2:1.

A hybrid approach combining manual annotation and threshold segmentation was employed to generate fracture labels. The original three-channel images were first converted to 8-bit single-channel format for binarization processing. Optimal segmentation thresholds were manually determined for each image during thresholding.

The initial threshold-generated images required substantial refinement due to white edges caused by identical pixel values at image borders and fracture boundaries. These artifacts were manually corrected by filling edges with black. Additional noise removal was performed using color filling techniques, followed by detailed verification through comparison between original images and preliminary labels to ensure annotation accuracy. The final labels underwent rigorous quality control to maintain segmentation precision.

The final labels underwent rigorous quality control to maintain segmentation precision. High-quality domain-specific datasets are fundamental to reliable model training, as demonstrated by Zheng et al. (2025) in constructing the HKCoral benchmark dataset. They implemented refined annotation and strict quality control to address the complexity of dense coral growth forms in wild environments^32^, a methodology aligned with our approach. For coal CT images with grayscale overlap and sparse microfractures, we adopted a hybrid annotation strategy combining manual correction of edge artifacts and noise removal, ensuring the accuracy and reliability of fracture labels to support effective model training.

In terms of data augmentation, images and their corresponding masks are randomly rotated by ± 90° and flipped horizontally and vertically. Additionally, a random rotation of ± 15° is applied again when the dataset is loaded into the model during each training batch to enhance the model’s robustness.

For normalization, to facilitate the verification and inspection of dataset accuracy, the grayscale values of masks are set to binary values (0 and 255) during mask creation—where coal matrix, background, and other non-target regions are assigned 0, and fractures are assigned 255. When loading data, the masks are further normalized from the grayscale range [0, 255] to [0, 1] (with coal matrix and background as 0, and fractures as 1) to ensure compatibility with model training.

In the dataset loading part of the model, The Image is scaled by BICUBIC interpolation (Image.BICUBIC). The label mask is scaled using the NEAREST neighbor interpolation (Image.NEAREST). Both are scaled to 512*512 to reduce the memory footprint during training.

### Experimental environment and parameter settings

The training environment for this study was a computing platform running Ubuntu 24.04.2 LTS, with an Intel^®^ Xeon^®^ W-3265 processor, 512 GB of RAM, and an NVIDIA GeForce RTX 4090 GPU. The programming language used was Python 3.13.5, and the deep learning framework was PyTorch 2.6.0 + cu129.

All comparative models were trained using the same set of hyperparameters: a batch size of 8, a learning rate of 0.0001, and training for 100 epochs, ensuring adequate convergence for all models.

### Evaluationmetrics

To scientifically validate the comprehensive performance of the proposed model in real-world coal industrial scenarios, this section establishes a complete quantitative evaluation system, conducting a thorough analysis from three dimensions: segmentation accuracy, computational efficiency, and resource consumption. This system includes not only traditional segmentation accuracy metrics such as Pixel Accuracy (PA), Intersection over Union (IoU), and Dice coefficient but also introduces key efficiency metrics like Parameter count (Params), Computational complexity (FLOPs), and Inference latency (Latency), addressing the practical deployment requirements in underground coal mines. These multi-dimensional quantitative assessments objectively measure whether the model, while maintaining high segmentation accuracy, meets the stringent computational constraints of mining intrinsic safety equipment, providing a reliable performance benchmark and decision basis for algorithm deployment in the field of intelligent coal mining.

1. Pixel Accuracy (PA)​​ measures the overall accuracy of pixel-level classification, calculated as:
  8 $$\:PA=\frac{\sum\:_{i=0}^{C}({TP}_{i}+{TN}_{i})}{\sum\:_{i=0}^{C}({TP}_{i}+{FP}_{i}+{FN}_{i}+{TN}_{i})}$$
  where TP, TN, FP, and FN represent True Positives, True Negatives, False Positives, and False Negatives, respectively.
  Mean Pixel Accuracy (mPA) is the average PA of all categories (fracture and background in this study), reflecting the overall classification accuracy across different targets.
2. ​Crack Intersection over Union (Crack IoU)​​ is a core metric for evaluating the spatial overlap accuracy of fracture segmentation, calculated as:
  9 $$\:Crack\:IoU=\frac{{TP}_{crack}}{{TP}_{crack}+{FP}_{crack}+{FN}_{crack}}$$
  Mean Intersection over Union (mIoU) is the average IoU of all categories, serving as a comprehensive metric for global segmentation performance.
3. ​Crack Dice Coefficient (Crack Dice)​​ is a key metric for measuring the overlap of fracture regions, calculated as:
  10 $$\:{Dice}_{crack}=\frac{2\times\:{TP}_{crack}}{2\times\:{TP}_{crack}+{FP}_{crack}+{FN}_{crack}}$$
4. ​Crack Recall​ assesses the model’s ability to detect true fractures, calculated as:
  11 $$\:{Recall}_{crack}=\frac{{TP}_{crack}}{{TP}_{crack}+{FN}_{crack}}$$
5. Crack Precision​ controls the false positive rate in fracture segmentation, calculated as:
  12 $$\:{Precision}_{crack}=\frac{{TP}_{crack}}{{TP}_{crack}+{FP}_{crack}}$$
6. Model Parameters (Params)​​ is a key factor determining hardware deployment feasibility. For a model, it’s the sum of all trainable parameters:
  13 $$\:Params=\sum\:_{l=1}^{L}({K}_{h}^{\left(l\right)}\times\:{K}_{w}^{\left(l\right)}\times\:{C}_{in}^{\left(l\right)}\times\:{C}_{out}^{\left(l\right)}+{C}_{out}^{\left(l\right)})$$
7. Computational Complexity (FLOPs)​​ reflects the computational burden during model inference, typically calculated as the total number of floating-point operations required for a single forward pass of a fixed-size input:
  14 $$\:FLOPs=\sum\:_{l=1}^{L}\left[2\times\:{H}_{l}\times\:{W}_{l}\times\:\left({K}_{h}^{\left(\left(l\right)\right)}{K}_{w}^{\left(\left(l\right)\right)}{C}_{i}{n}^{\left(\left(l\right)\right)}+{C}_{i}{n}^{\left(\left(l\right)\right)}{C}_{o}u{t}^{\left(\left(l\right)\right)}\right)\right]$$

GFlops (Giga FLOPs) refers to 10^9^ floating-point operations, used to quantify the computational load in a more intuitive scale.

### Results and analysis

To evaluate the proposed model’s advantages in reducing computational complexity, parameter count, and segmentation accuracy, detailed ablation experiments, five-fold cross-validation experiments, and comparative experiments with state-of-the-art models in the field were designed. All experiments were independently repeated three times, with final results based on the average value (standard deviation < 1.5%).

#### Model ablation study

Ablation experiments were conducted to verify the effectiveness of the three core optimization mechanisms of LightECA-UNet: Depthwise Separable Convolution (DSC), ECA Attention Mechanism (ECA), and Channel Reduction (CR). Eight combinations including the basic UNet were tested, using the evaluation metrics established in Sect. 2.3 to comprehensively measure segmentation accuracy and lightweight performance. Detailed results are shown in Table 1.

**Table 1:** Comparison of metrics in the model ablation study.

Methods	PA	mPA	mIoU	Crack IoU	Crack Dice	GFlops	Params/M
LightECA-Unet	99.46	98.53	97.08	94.38	97.3	6.01	**0.55**
Basic UNet	97.71	94.72	88.51	79.95	88.59	218.65	31.04
UNet-DSC	96.51	92.72	87.56	77.38	87.75	32.99	2.18
UNet-ECA	**99.55**	**98.78**	97.38	**94.78**	**97.67**	220.34	31.31
UNet-CR	97.51	94.61	87.34	74.64	87.54	130.24	13.33
UNet-DSC-ECA	99.53	98.76	**97.48**	94.68	97.58	33.03	2.18
UNet-DSC-CR	96.35	92.23	86.54	73.84	86.78	**5.99**	**0.55**
UNet-ECA-CR	99.51	98.64	97.35	94.65	97.55	130.84	13.33

Values in bold signify the optimal performance metrics among all compared methods.

As shown in Table 1, the basic UNet, which uses standard double convolution and fixed channel configuration, has a computational load of 218.65 GFlops and 31.04 M parameters, with a Crack IoU of only 79.95%. Models incorporating ECA (UNet-ECA, UNet-DSC-ECA, UNet-ECA-CR) achieved significantly improved segmentation metrics, with UNet-ECA reaching a Crack IoU of 94.78%, confirming ECA’s effectiveness in enhancing low-contrast fracture recognition. Models adopting DSC and CR (UNet-DSC, UNet-CR, UNet-DSC-CR) achieved significant lightweight effects, with UNet-DSC-CR reducing computational load to 5.99 GFlops and parameters to 0.55 M, but their segmentation accuracy was severely degraded (Crack IoU ≤ 77.38%). The proposed LightECA-UNet, integrating all three mechanisms, achieved an optimal balance: maintaining the lightweight advantage of 6.01 GFlops and 0.55 M parameters while achieving a Crack IoU of 94.38% and Crack Dice of 97.3%, which is 14.43% points higher than the basic UNet. Additionally, comparing models with and without CR (e.g., UNet vs. UNet-CR, UNet-DSC vs. UNet-DSC-CR) shows that channel reduction can reduce computational load and parameters by more than 50% with negligible accuracy loss, verifying the feasibility of the adaptive channel pruning strategy.

#### K-fold cross-validation

**Fig. 4:**
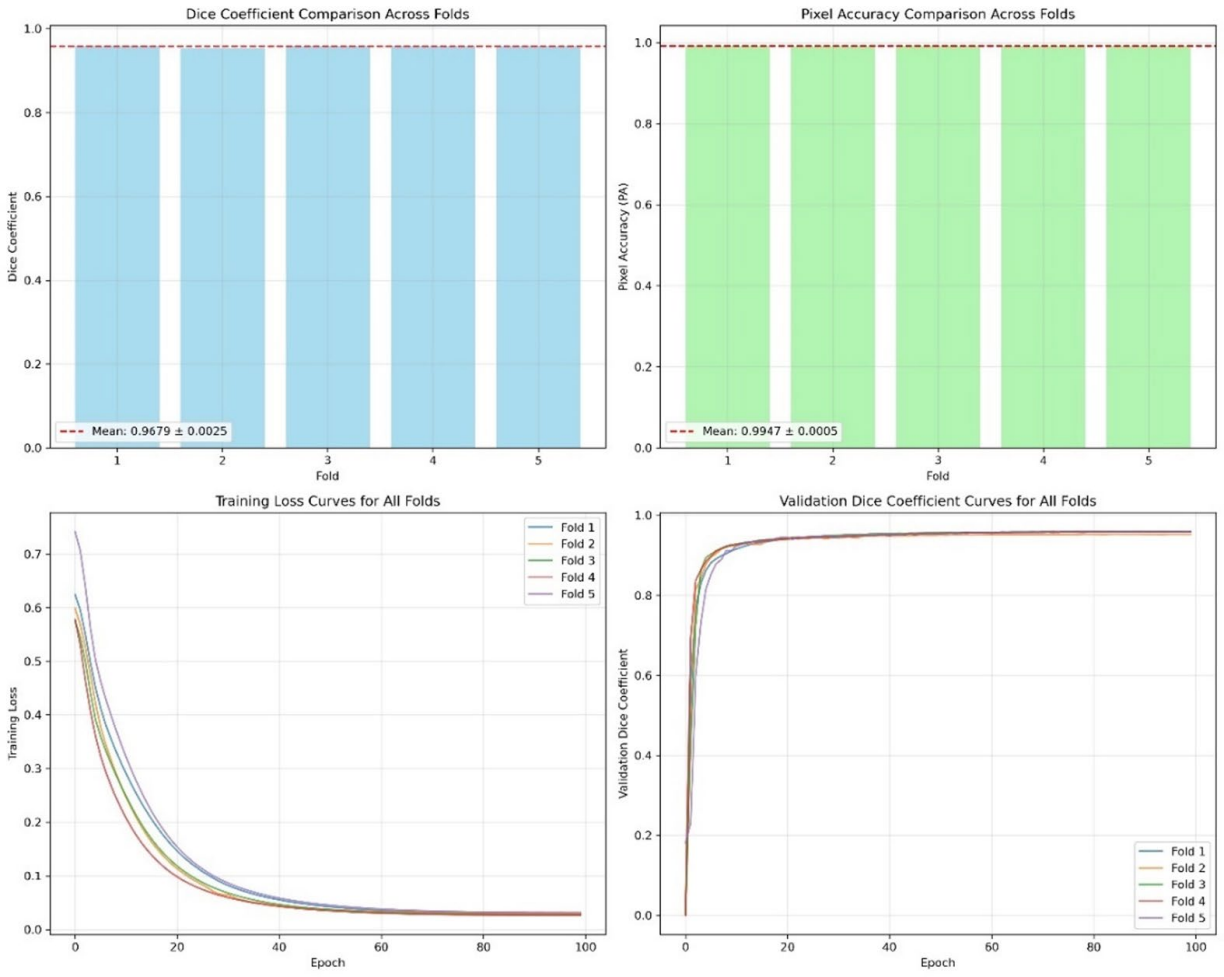
Training process parameter log of the five-fold cross-validation experiment.

To rigorously evaluate the generalization ability of the proposed model, and considering the scarcity of publicly available coal CT fracture image datasets, we conducted comprehensive five-fold cross-validation on our own CT dataset. K-fold cross-validation is widely recognized as a reliable method for estimating model performance and mitigating overfitting risks, with Rodriguez et al. (2010) verifying through sensitivity analysis that it enables stable prediction error estimation when applied appropriately^33^. The experimental results are shown in Table 2; Fig. 4.

**Table 2:** Results of the five-fold cross-validation experiment.

	PA	mPA	mIoU	Crack IoU	Crack dice	Crack precision	Crack recall	GPU time/ms
Fold 1	99.47	98.33	96.81	94.23	96.95	97.32	97.22	3.6192
Fold 2	99.42	98.03	96.35	93.42	96.31	96.63	96.51	3.6089
Fold 3	99.52	98.52	97.09	94.78	96.93	97.25	97.39	3.6573
Fold 4	99.47	98.45	96.98	94.05	96.92	97.01	96.87	3.6089
Fold 5	99.46	98.29	96.67	93.89	96.86	96.89	96.99	3.6427
Average	99.47 ± 0.05	98.31 ± 0.28	96.72 ± 0.45	94.11 ± 0.81	96.79 ± 0.25	96.97 ± 0.39	96.95 ± 0.53	3.6140 ± 0.0552

The test results show that the Dice coefficient is 96.79% ± 0.25%, and the pixel accuracy is 99.47% ± 0.05%. The stable segmentation performance across different data splits confirms the model’s robustness and mitigates potential overfitting issues.

#### Comparison with leading industry models

**Fig. 5:**
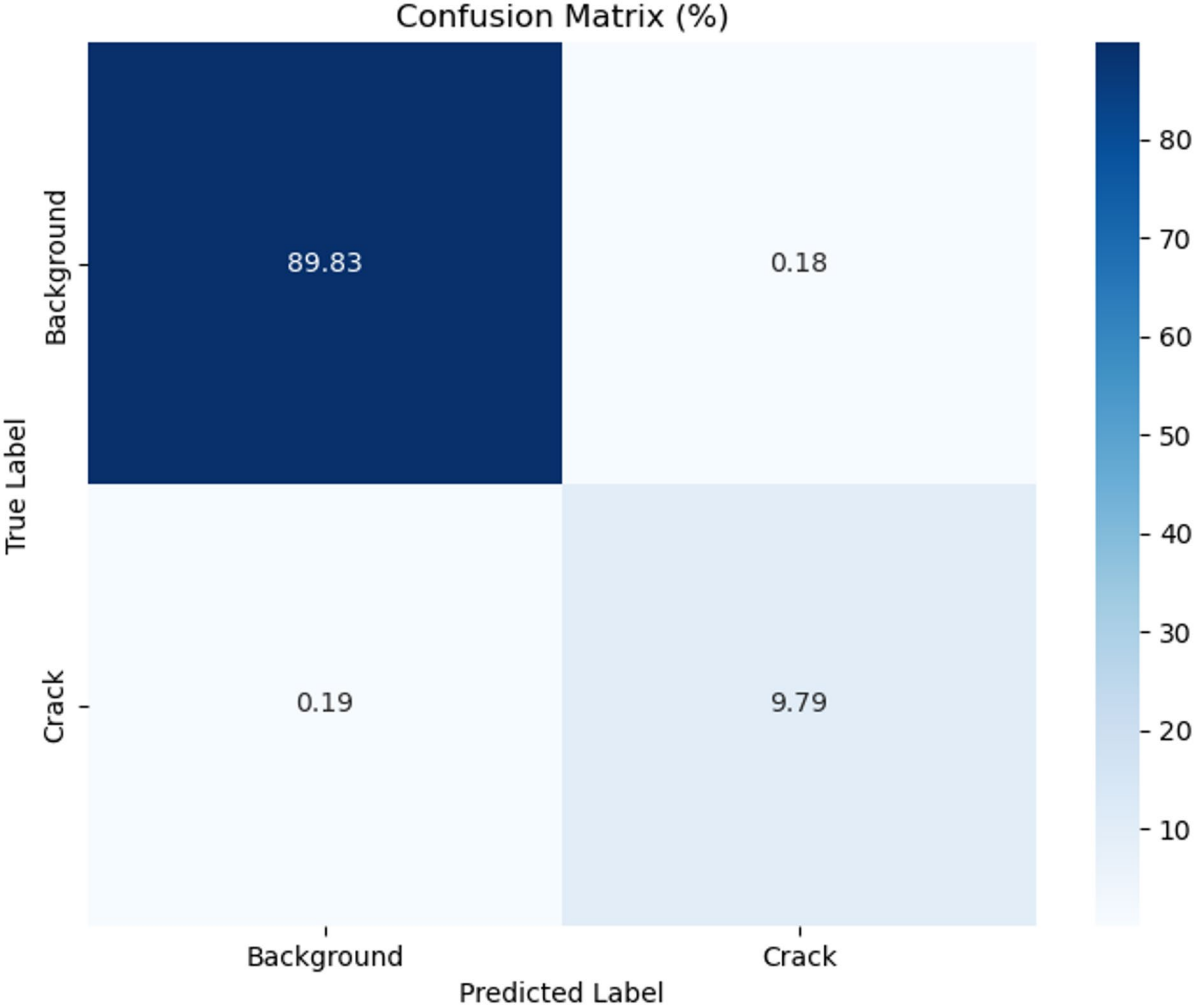
Confusion matrix.

**Fig. 6:**
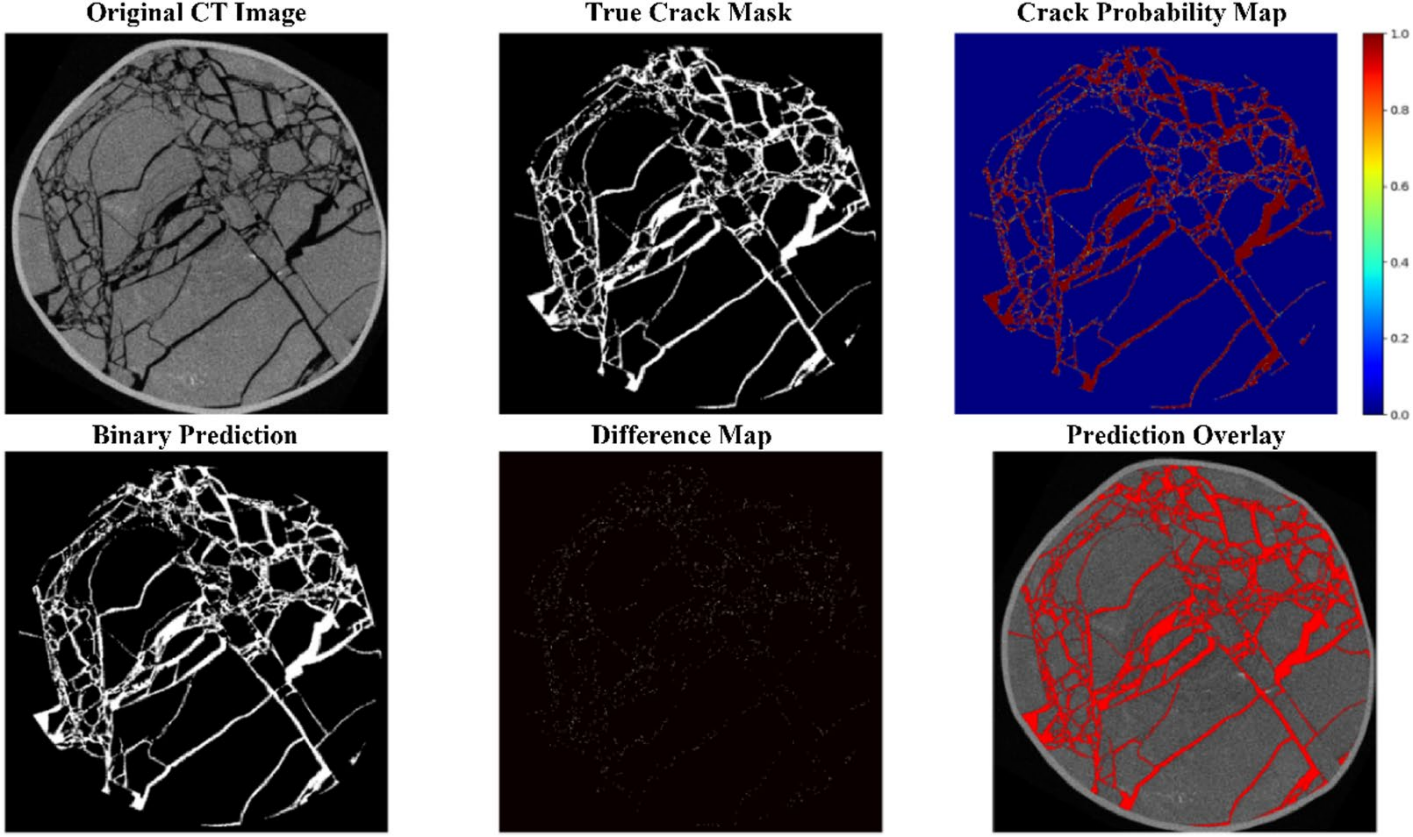
Specific segmentation results.

**Fig. 7:**
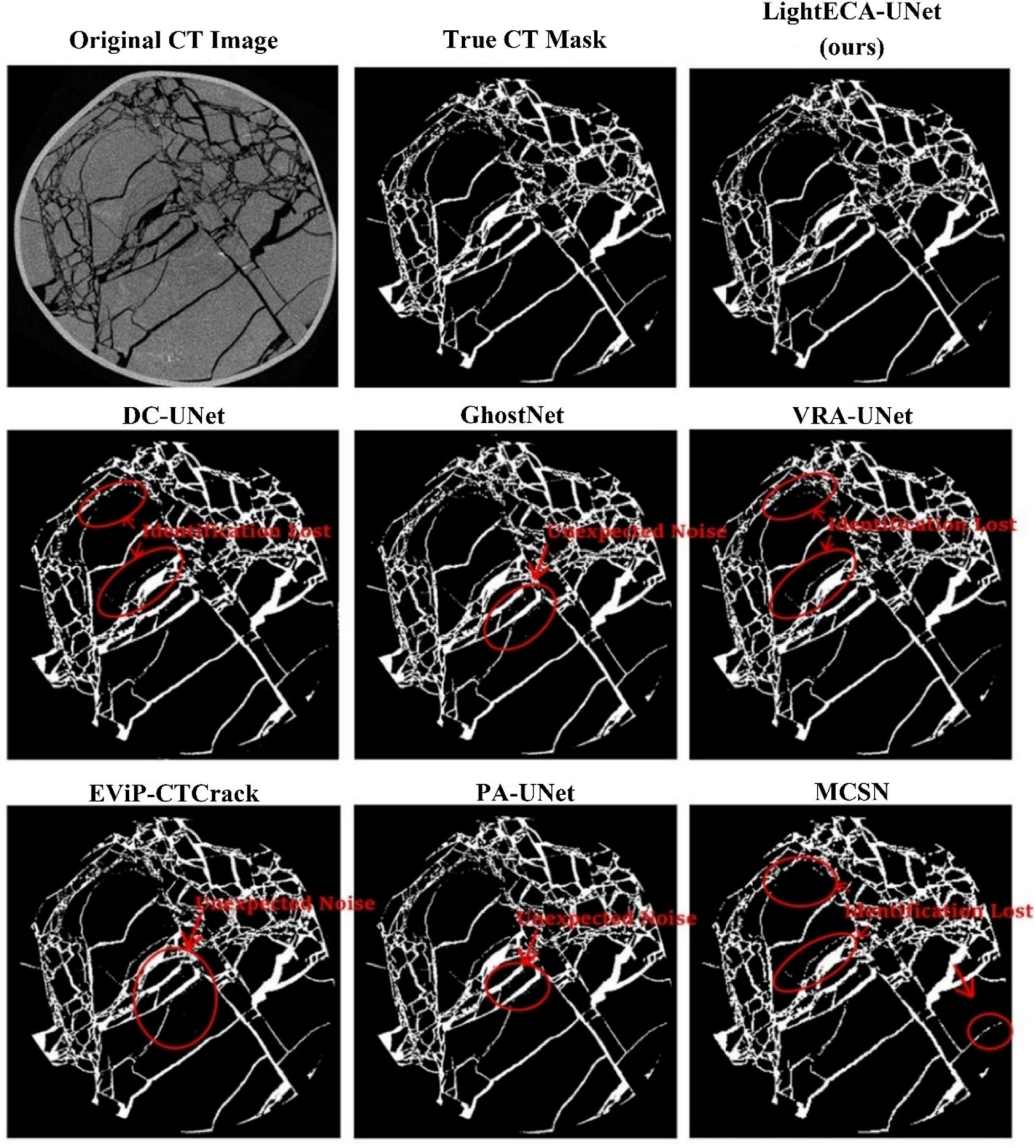
Comparison of segmentation results with industry-leading models.

Comparative experiments were performed with six advanced models from the industry, namely DC-UNet, GhostNet, VRA-UNet, EViP-CTCrack, PA-UNet and MCSN, to validate the comprehensive performance of LightECA-UNet. All models were trained with identical hyperparameters on the enhanced dataset. GPU inference time was measured using an NVIDIA RTX 4090 24G graphics card, with the measurement excluding the time consumed by model loading and result readout. Results of the computational load and parameter comparison are presented in Table 3, while segmentation metric comparison results are shown in Table 4. Visual comparisons of segmentation results are provided in Figs. 5, 6 and 7.

**Table 3:** Comparison of computational load and parameters with leading industry models.

Metrics	LightECA-UNet	DC-UNet		GhostNet	VRA-UNet	EViP-CTCrack	PA-UNet	MCSN
GFlops	**6.01**	46.03		79.83	220.81	197.24	167.43	102.92
Params/M	**0.55**	4.22		8.11	53.43	29.48	27.04	28.26
GPU time/ms	**3.61**	4.67		5.39	5.34	6.01	4.95	4.42

The bolded entries are used specifically to highlight the performance of our proposed model (LightECA-UNet) to distinguish it from the baseline models.

**Table 4:** Comparison of segmentation metrics with leading industry models.

Methods	PA	mPA	mIoU	Crack IoU	Crack dice	Crack precision	Crack recall
LightECA-UNet	**99.46**	**98.53**	**97.08**	**94.38**	**97.3**	**97.23**	**97.38**
DC-UNet	98.58	96.15	92.61	86.78	92.92	92.74	93.1
GhostNet	99.11	97.62	95.27	91.52	95.57	95.4	95.74
VRA-UNet	98.53	96.28	92.41	86.43	92.72	91.99	93.46
EViP-CTCrack	99.14	97.75	95.41	91.76	95.72	95.38	96.02
PA-UNet	99.17	97.08	95.54	92.01	95.83	95.58	96.09
MCSN	97.32	93.59	86.94	76.83	86.91	84.95	88.93

The bolded entries are used specifically to highlight the performance of our proposed model (LightECA-UNet) to distinguish it from the baseline models.

Table 3 illustrates that LightECA-UNet achieves the lowest computational load and parameter count among all compared models, with 6.01 GFlops of computational complexity and 0.55 million parameters. Models without lightweight design, including VRA-UNet, EViP-CTCrack and PA-UNet, exhibit computational loads 32.8 to 36.7 times higher than LightECA-UNet and parameter counts 25.8 to 53.4 times higher. Even among lightweight counterparts, DC-UNet and GhostNet still have higher resource consumption. DC-UNet has a computational load of 46.03 GFlops and 4.22 million parameters, while GhostNet has 79.83 GFlops of computational complexity and 8.11 million parameters. Their computational loads are 7.7 to 13.3 times higher than LightECA-UNet, and their parameter counts are 7.7 to 14.7 times higher. In terms of inference efficiency, LightECA-UNet achieves an average GPU inference time of 3.61 ms, which is 0.8 to 2.4 ms faster than the other compared models.

Regarding segmentation accuracy, as presented in Table 4, LightECA-UNet outperforms all comparative models across key evaluation metrics, including a Crack IoU of 94.38%, a Crack Dice coefficient of 97.3% and a Crack Precision of 97.23%. DC-UNet and MCSN both adopt depthwise separable convolution but lack effective attention compensation mechanisms, leading to Crack IoU values 7.6 and 17.55% points lower than LightECA-UNet respectively. GhostNet was originally designed for image classification tasks, resulting in poor adaptability to coal CT image scenarios and a Crack IoU 2.86% points lower than LightECA-UNet. VRA-UNet, EViP-CTCrack and PA-UNet suffer from insufficient module synergy, with their Crack IoU values ranging from 2.37 to 7.95% points lower than LightECA-UNet. Visual segmentation results, as shown in Fig. 7, further confirm the superiority of LightECA-UNet. It can segment blurred fractures and micro-fractures more accurately, with fewer false positive and false negative predictions compared to the other models.

## Discussion

### Mechanism analysis of model performance advantages

LightECA-UNet achieves an excellent balance between lightweight performance and high accuracy through the efficient synergy of depthwise separable convolution, efficient channel attention and adaptive channel pruning. Traditional lightweight models typically encounter the challenge of accuracy degradation when reducing computational load, a problem that LightECA-UNet addresses through targeted structural optimization. Depthwise separable convolution decomposes traditional convolution into depthwise and pointwise operations, reducing computational load to approximately one-ninth of that of traditional convolution. However, its inherent channel isolation issue weakens feature interaction. The efficient channel attention mechanism compensates for this limitation perfectly by modeling local cross-channel interactions through one-dimensional convolution without dimensionality reduction. Different from SE and CBAM attention mechanisms, efficient channel attention only models sparse relationships between adjacent channels. This characteristic ensures high compatibility with the low-intensity and high-sparsity fracture features output by depthwise separable convolution, while avoiding overfitting and noise amplification caused by global feature fusion. This symbiotic structure of efficient channel attention and depthwise separable convolution enables the model to maintain strong feature extraction capabilities while reducing computational complexity.

Adaptive channel pruning further optimizes the model’s resource footprint without compromising accuracy. The adjustment of the encoder-decoder channel configuration is based on the statistical characteristics of coal CT images. The encoder adopts a channel configuration of 32–64-128–256-256, and the decoder uses a symmetric 256-128-64-32-32 configuration. Coal CT images feature narrow fracture grayscale variation, eliminating the need for high-dimensional feature spaces. This scenario-specific pruning strategy avoids the parameter redundancy of the basic UNet’s fixed 64–128-256–512 configuration, reducing the number of parameters from 31 million to 550,000. Ablation experiments verify that this adjustment has a negligible impact on accuracy, demonstrating that the optimized channel configuration fully meets the feature representation requirements of coal fracture segmentation.

In contrast, the performance shortcomings of comparative models stem from unreasonable structural design. The dual attention mechanism of DC-UNet relies on inter-channel interaction, which is weakened by depthwise separable convolution. This leads to insufficient saliency of spatial attention maps and high misjudgment rates for blurred fractures. The VGG16 backbone of MCSN is incompatible with depthwise separable convolution. The dense and redundant features of VGG16 are disrupted by the channel independence of depthwise separable convolution, and the introduced atrous convolution forms an information-sparse receptive field that reduces feature utilization. The G-bneck structure of GhostNet has higher computational complexity than depthwise separable convolution. The stacking of dual Ghost modules and SE attention further increases the computational load, preventing the achievement of optimal lightweight effects. These results confirm that lightweight design for coal CT fracture segmentation must be scenario-adaptive, integrating lightweight operations with targeted attention mechanisms to balance efficiency and accuracy.

### Implications for engineering applications

The high-precision and lightweight nature of LightECA-UNet offers significant advantages for practical coal mining and rock mechanics research.

In terms of engineering applications for real-time on-site Monitoring, Coal fracture analysis is increasingly required to be performed at the mining face or in field laboratories. Existing heavy-duty deep learning models require expensive GPU clusters, which are difficult to deploy in resource-constrained mining environments. As a Lightweight Model, LightECA-UNet’s 0.55 million parameters and 3.61 millisecond inference time significantly reduces the demand for FLOPs and memory, which meet on-site real-time application. This facilitates the integration of our algorithm into portable CT scanning devices or edge computing terminals, enabling rapid, on-site characterization of coal mass stability during excavation.

In terms of quantitative analysis of coal reservoir permeability, accurate coal fracture segmentation serves the prerequisite for calculating the equivalent permeability and porosity of coal seams. Traditional models often suffer from “fracture disconnection” in low-contrast CT regions, which leads to a massive underestimation of fluid conductivity in numerical simulations. By utilizing the LightECA mechanism, the proposed model maintains the topological continuity of micro-fractures. From an engineering standpoint, this ensures more reliable input data for gas drainage and CO2 sequestration modeling, directly impacting the safety and efficiency of underground operations. And the model enhanced mIoU, thereby reducing topological errors in seepage simulation and ultimately improving the prediction accuracy of gas extraction.

In terms of enhancing the accuracy of rock mass stability assessment, the identification of fine cracks and their spatial distribution is crucial for predicting coal and gas outbursts. The improved segmentation of crack tips and intersections provided by the model allows engineers to better quantify the damage variables of the coal matrix. Consequently, this provides a more scientific basis for determining the optimal locations for hydraulic fracturing or pressure relief drilling.

Beyond these domain-specific advances, the scenario-adaptive lightweight design paradigm proposed in this study provides a new reference for low-contrast, small-target segmentation tasks in geoscientific imaging. Beyond coal fracture segmentation, it can be extended to related research fields such as rock mass crack segmentation in tunnel engineering and micro-pore segmentation in shale, promoting the application and innovation of lightweight deep learning technology in geoscientific data processing. Accurate fracture segmentation data also opens up new research directions such as multi-scale fracture network reconstruction and dynamic prediction of fracture evolution during mining, further expanding the depth and breadth of coal-related interdisciplinary research.

## Conclusion

To address the core challenges of inadequate segmentation accuracy and poor feasibility of mine-edge deployment in coal CT image fracture segmentation, this study proposes a lightweight segmentation model named LightECA-UNet. The model integrates depthwise separable convolution, efficient channel attention, and adaptive channel pruning, realizing dual optimization of segmentation accuracy and computational efficiency for coal fracture segmentation tasks.

Experimental results demonstrate that LightECA-UNet achieves a Crack IoU of 94.38% and a Crack Dice coefficient of 97.3%. Compared with the basic UNet, these metrics are improved by 14.43 and 8.71% points respectively. Among state-of-the-art segmentation models, LightECA-UNet exhibits the lowest computational load and parameter count, with 6.01 GFlops of computational complexity, 0.55 million parameters, and an inference time of 3.61 ms. Such performance characteristics enable the model to be deployed on mine intrinsic safety equipment with strict resource constraints. Five-fold cross-validation results further confirm the model’s strong robustness and low overfitting risk, ensuring its reliable performance in practical applications.

The core innovations of this study, including the ECA-DSC symbiotic block and adaptive channel pruning strategy, provide an effective scenario-adaptive lightweight design paradigm for coal CT image fracture segmentation. With its high-precision and efficient segmentation capabilities, LightECA-UNet offers reliable technical support for coal structure quantitative analysis, mine safety early warning, and intelligent mining practices. This work promotes the engineering application of deep learning technologies in the coal mining industry, laying a foundation for the quantitative and intelligent development of coal structure evaluation.

Notably, this study has certain limitations. The performance of LightECA-UNet has only been validated on CT images of a single coal type. Future research will focus on verifying its adaptability to complex coal scenarios, integrating advanced data augmentation techniques, and optimizing the model for 3D coal fracture segmentation.

## Data Availability

Data sets generated during the current study are available from the corresponding author on reasonable request.
